# Influence of the Camera Viewing Angle on OpenPose Validity in Motion Analysis

**DOI:** 10.3390/s25030799

**Published:** 2025-01-28

**Authors:** Melanie Baldinger, Lara Marie Reimer, Veit Senner

**Affiliations:** 1 School of Engineering and Design, Technical University of Munich, 85748 Garching b. Munich, Germany; 2 Institute for Digital Medicine, University Hospital Bonn, 53127 Bonn, Germany; lara.reimer@ukbonn.de

**Keywords:** human motion capture, mobile motion capture, markerless motion capture, human pose estimation, OpenPose, camera angle, validity, joint angles

## Abstract

(1) Background: With human pose estimation on the rise in the field of biomechanics, the need for scientific investigation of those algorithms is becoming evident. The validity of several of those algorithms has been presented in the literature. However, there is only limited research investigating the applicability of human pose estimation outside the lab. The aim of this research was to quantify the effect of deviating from the standard camera setup used in biomechanics research. (2) Methods: Video data from four camera viewing angles were recorded and keypoints estimated using OpenPose. Kinematic data were compared against a gold-standard marker-based motion capture system to quantify the effect of the camera viewing angle on the validity of joint angle estimation of the knee, hip, elbow and shoulder joints. (3) Results: The results of this study showed reasonable correlations between the joint angles of OpenPose and the gold standard, except for the shoulder. However, the analysis also revealed significant biases when comparing the joint angles inferred from the different viewing angles. In general, back-viewing cameras performed best and resulted in the lowest percental deviations. (4) Conclusions: The findings of this study underscore the importance of conducting a detailed examination of individual movements before proposing specific camera angles for users in diverse settings.

## 1. Introduction

Human pose estimation (HPE) is an emerging field in the domain of biomechanics. It employs computer vision methods for automatic tracking of so-called keypoints, or anatomical landmarks, of human bodies from images or video data. The purpose is to identify the position and orientation of body segments and hence describe the subject’s pose by estimating joint centers [1]. HPE algorithms are mostly trained on extensive image and video datasets containing labeled human poses [2,3].

Human motion analysis is an essential tool in the study of movements in biomechanics, sport science and rehabilitation. Any kind of human movement can be quantified, analyzed and optimized using human body kinematics derived from the estimation of the position and orientation of body parts. Motion capture (MoCap) technologies can be employed to address a range of questions pertinent to sports performance analysis [4], such as technique evaluation [5], monitoring athletes’ improvement over time, injury prevention [6] or other sports-specific questions [7]. Clinical applications of this technology include, for example, gait analysis [8,9] and balance assessment [10,11], as well as the long-term monitoring of rehabilitation [12]. Stenum et al. [13] present an overview of applications of HPE across the lifespan, ranging from developmental tracking over performance optimization and injury prevention to clinical examination of persons with neurological damage or diseases. In biomechanics, accurate human joint angle estimation is essential for meaningful analysis. Highly accurate human body kinematics are of great importance, for example, when differentiating between healthy and pathological movement patterns, when monitoring recovery progression or when assessing risk of injury.

The current gold standard for MoCap employs an optical marker-based approach, wherein the subject is equipped with artificial color contrasting markers on predetermined anatomical landmarks. Movements of the subject are recorded using two or more high-precision and usually high-speed cameras. Those cameras need to be synchronized and calibrated within a certain movement space. Markers are then tracked automatically or manually using specialized software tools. Human body models incorporated in these tools allow for sophisticated kinematic analysis. This is an elaborate approach requiring expert knowledge and it is typically restricted to laboratory settings within a limited area of movement. Measurement accuracy highly depends on the correct positioning of the markers [14], which is a time-consuming procedure requiring an experienced operator. Additionally, it is necessary to maintain constant lighting conditions, which can prove challenging in field settings or everyday training environments. Consequently, the measurement is typically confined to indoor environments. This excludes a huge variety of movements and sports that cannot be reproduced in a laboratory setting, such as, for example, skiing or field sports. These challenges suggest that subjects do not move in a natural environment, and it is questionable if movement execution is unbiased [14,15].

HPE, however, is able to compensate for some of the drawbacks of the traditional marker-based MoCap used as the gold standard in biomechanics and rehabilitation. Videos can be recorded in an unobtrusive and non-invasive way in a variety of environments. It even allows the use of smartphones in home environments for recording of training or rehabilitation progression.

The advances in computer vision have paved the way for many HPE algorithms to be developed within the last years [8,16]. A prominent HPE algorithm used in biomechanics research is OpenPose [17]. It is an open-source software [18] for multi-person 2D pose estimation. The estimation of human body parts in an image or video is based upon a multi-stage deep learning architecture using so-called Part Affinity Fields (PAFs), which represent 2D vector fields that contain information about the location and orientation of certain body parts. The algorithm is able to detect up to 135 keypoints of the human body, foot and hand as well as facial keypoints depending on the model used [17]. Another example of an HPE algorithm is AlphaPose [19]. The algorithm is composed of a five-stage pipeline including detection modules, transformation modules and pose estimation modules. The estimation results in up to 136 keypoints, of which 20 are for the body, while the rest are keypoints of the feet, hands and face. BlazePose [20] is a further example of a lightweight human pose estimator. Thirty-three keypoints can be detected using a so-called detector–tracker setup. The body pose detector identifies the pose region-of-interest within an image followed by a pose tracker network to estimate the keypoints [20]. Further common algorithms in human movement sciences along with the aforementioned include DeeplabCut, PoseNet, HRNet, MediaPipe, EfficientPose and MoveNet [8].

A variety of researchers have investigated the validity of OpenPose and compared it against the gold-standard marker-based MoCap system in different scenarios and use cases: gait analysis [21,22,23,24,25], squats [26,27], walking and running [28,29,30], cycling [5,31], the drop vertical jump test [32], taekwondo [33] and other athletic movements [34].

Validation studies typically focus on sagittal and frontal camera angles since these angles usually produce more reliable 2D joint angle data in most contexts. This results in a lack of adaptability to varying environments and applications. While sagittal and frontal views are the most established camera angles in research, other camera angles are often used in real-world settings due to space limitations or varied environments. The validity of those validation results is therefore limited to the conventional angles.

Limited literature exists that investigates the impact of deviating from those established camera angles [24,35]. Wang et al. [24] studied the effect of different camera angles and distances on the accuracy of gait parameters when validating OpenPose during gait analysis. They obtained statistically significant effects of the camera angle and distance on measurement accuracy [24]. Mehdizadeh et al. [35] examined two different camera angles when comparing the accuracy of three HPE algorithms (OpenPose, AlphaPose, Detectron) during gait. The cameras were mounted at different heights to mimic eye-level and ceiling-mounted, tilted-down viewing angles. Videos of participants walking were recorded from the front and the back. The results suggest no major difference between the two viewing angles as well as front and back views when validating gait variables against reference motion capture [35].

The impact of alternative camera angles has not been thoroughly investigated and quantified, therefore limiting the applicability in diverse setups, such as, for example, the home training environment [36,37] or even telerehabilitation [6]. Especially in environments and situations where no experts are available to provide guidance and feedback on the performed movements, it can be valuable to have a tool that helps with analyzing the movements. Examples of virtual coaches as proposed in the literature have been developed for use in home training [36] and in yoga [37]. Patients or trainees at home might lack an understanding of which angle yields the most reliable results. This research can shed light on how the camera angle influences accuracy and therefore can provide insights on how to minimize these effects, ensuring accurate tracking of patient or trainees’ movements. Moreover, Suo et al. [38] highlight in their most recent review the necessity of research addressing the use of motion capture technologies in practical sports scenarios and outdoors.

We, therefore, sought to systematically investigate and quantify the influence of the camera angle on the validity of OpenPose joint angle estimation. We set out to provide insights into potential inaccuracies when deviating from the standard camera angles. This research produced practical recommendations for researchers and practitioners on the ideal positioning of a single camera when estimating human poses using OpenPose. Through an understanding of how accuracy is influenced by different perspectives, users can optimize camera positioning in diverse settings, thereby enhancing the reliability of joint angle estimation. By addressing the impact of camera viewing angles on joint angle estimation, this study paves the way for more flexible and mobile motion analysis.

## 2. Materials and Methods

### 2.1. Participants

In total, 20 healthy participants (8 females and 12 males; age: 25.1 ± 5.0 years; height: 1.75 ± 0.09 m; weight: 70.9 ± 12.2 kg; and BMI: 23.11 ± 2.52) volunteered to take part in this study. All subjects were in good health without any orthopedic diseases or injuries. We anticipated that variations in weight, height and body type would not influence the results. Both measurement systems are designed to capture motion data in a manner that is largely independent of the subject’s body size or composition. Participants were instructed to wear close-fitting outfits for the experiment. All participants were informed about the experimental protocol upfront. Informed written consent was obtained from all subjects involved in this study.

This study was conducted in accordance with the Declaration of Helsinki and approved by the Ethics Committee of the Technical University of Munich (protocol code 2022-222-NP on 8 April 2022).

### 2.2. Experimental Setup

This study was performed in a laboratory equipped with 12 Vicon cameras (Vicon Motion Systems Ltd., Oxford, UK) for the optical marker-based MoCap. Reflective markers were attached to the subjects using the Vicon Full Body Plug-in Gait model [39]. The system was first calibrated using a Vicon calibration wand and then used to collect reference movement data. Static calibration was performed by recording the subject in a T-pose. The sampling frequency of the Vicon cameras was 200 Hz.

**Figure 1:**
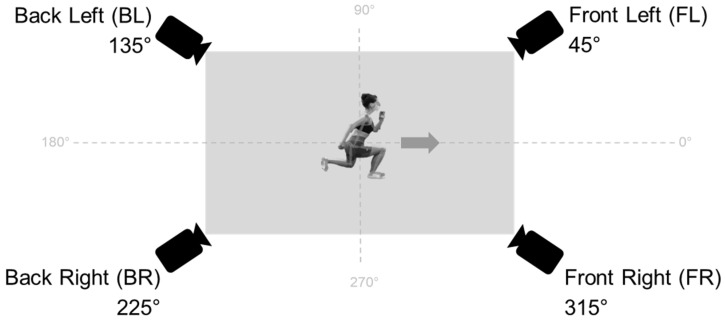
Camera setup. 4 iPad cameras are placed around the measurement space in 45° angle intervals.

To investigate the effect of the camera angle on kinematics derived from 2D human pose estimation, 4 RGB cameras (iPad Pro 11″, 2021 Model; Apple Inc., Cupertino, CA, USA) were positioned at 45° intervals around the measurement space. The camera setup and naming of the cameras are depicted in Figure 1. The subjects’ viewing and movement direction was at 0°, and cameras recorded those movements from the front left (FL), front right (FR), back left (BL) and back right (BR). Those camera angles were chosen for two reasons: (1) to examine the angles that fall between frontal and sagittal perspectives, and (2) to represent and investigate the impact in real-world scenarios where individuals in home or rehabilitation training may only have one (smartphone) camera available. This, and lack of experience in this data capture, may lead to an inability to discern between frontal and sagittal views, consequently leading to a preference for an angle that falls between these two positions. All iPads were mounted on tripods. Custom-developed software was used for the iPads to ensure synchronized video recording with the front left (FL) camera designated as the master and all other cameras (BL, BR, FR) as slaves. Video data were recorded at a resolution of 1194 × 834 pixels with a mean sampling frequency of 43 fps, which was subject to a slight variance (±2.6 fps) across all recorded videos. A 12 MP wide lens with an f/1.8 aperture was used. Lighting conditions were kept constant for all participants by closing the window blinds and using the same artificial light for all experiments.

During the experiment, each participant was instructed to perform 6 repetitions of front lunges. This exercise was selected to track a full-body movement with all major joints involved. The starting position is upright standing in a neutral position. The exercise starts with the descending phase, which involves stepping forward with one leg while lowering the body until the anterior knee is bent at approximately 90 degrees and the posterior knee is positioned just above the ground. The torso remains upright. In the ascending phase, the front leg pushes back to the starting position. The exercise then resumes with the contralateral movement. Following the correct execution of the exercise, it is anticipated that flexion will be observed in the hip, knee, shoulder and elbow joints, which are the joints under investigation.

### 2.3. Data Collection and Processing

Marker-based data were collected using Vicon Nexus software (version 2.10.3). We calculated the kinematics using the predefined pipeline. Gaps in marker tracking were manually filled using a combination of Spline Fill, Pattern Fill or Rigid Body Fill in the Vicon Nexus software. Data were exported in the C3D file format.

**Figure 2:**
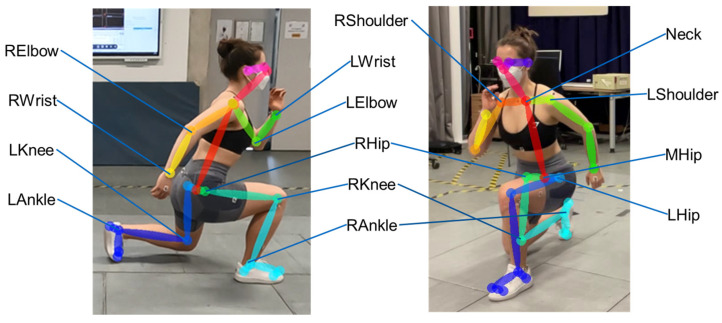
Definition of OpenPose keypoints used for calculating joint angles.

RGB video data were processed using OpenPose using the 25 keypoint model. The relevant keypoints for calculating knee, hip, shoulder and elbow joints are shown in Figure 2. OpenPose outputs a video file with an overlay of the estimated human pose (as can be seen in Figure 2) and a JSON file for each frame containing 2D coordinates of each of the 25 keypoints. Those coordinates were used to calculate the joint angles for knee flexion/extension, hip flexion/extension, shoulder flexion/extension and elbow flexion/extension. In order to facilitate the comparison of the OpenPose joint angles, the corresponding joint angle of the marker-based MoCap system in the respective plane, e.g., hip flexion/extension angle, was used. The definitions of the joint angles, their respective movement planes and how they were calculated using the OpenPose keypoints can be found in Table 1.

The following equation was used to calculate the angle (*θ*) between the two vectors ($\vec{a}$, $\vec{b}$), each representing a human body part (as described in Table 1):

(1) $$\theta =\arccos\left(\frac{\vec{a}\circ \vec{b}}{\left|\vec{a}\right|\circ \left|\vec{b}\right|}\right)$$

**Figure 3:**
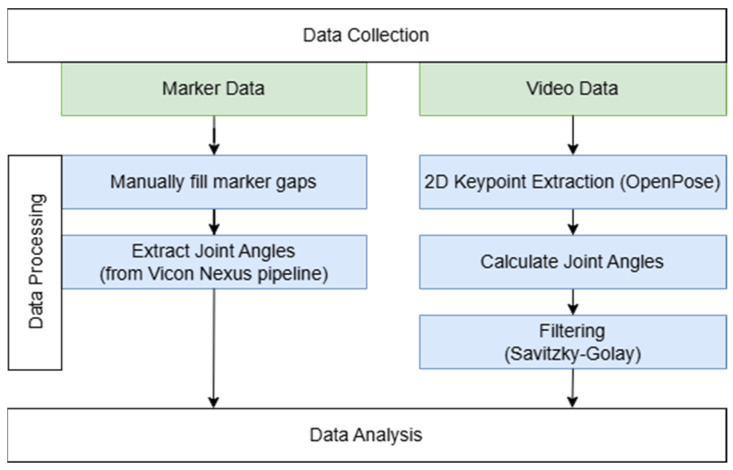
Flowchart of data processing for both marker-based and markerless MoCap systems.

Joint angle data from OpenPose were subsequently filtered using the Savitzky–Golay filter (third order with a window length of 15). This filter method was chosen to smooth the noisy components of the data, originating from the keypoint detected, which is a pixel value derived before calculating the joint angle, yet still preserving the peaks and valleys of the original data. For synchronization, we used the first and last peaks of the right knee angle of the 6 lunges recorded. The sampling frequency was then downsampled to the lower frequency in order to calculate statistical parameters. Data processing was performed using a custom Python script (Python version 3.10) and the Kinetics Toolkit [40]. The flowchart for the data processing is depicted in Figure 3 for both input data streams.

### 2.4. Statistical Analysis

First, we determined the correlation between each joint angle calculated and estimated from each of the camera viewing angles and the marker-based MoCap system as the gold standard using the correlation coefficient (r). The correlation coefficients were interpreted as small for values between 0.1 and 0.3, medium for values between 0.3 and 0.5 and large correlations for those greater than 0.5 [41]. Additionally, we calculated the intraclass correlation coefficients (ICCs). ICC(2,1) was obtained to assess absolute agreement and ICC(3,1) for consistency between the joint angles measured using the gold standard and all 4 camera viewing angles. ICC values were interpreted as poor (<0.5), moderate (0.5–<0.75), good (0.75–<0.9) and excellent (≥0.9) [42]. Root mean square error (RMSE) and bias were reported as a measure of systematic deviation. Furthermore, agreement was assessed using Bland Altman analysis. Finally, we investigated peak joint angle values. Therefore, we extracted joint angles at the peak knee flexion for each repetition of lunges, which represents the turning point of the movement and full range of motion for all four joint angles of interest. Statistical analysis was performed using IMB SPSS Statistics version 29.

## 3. Results

Data of four of the participants had to be excluded since a portion of the data was missing for technical reasons, with those reasons being marker visibility in three cases and no recording of the iPads in one case. Given that the excluded datasets were attributable to technical issues, namely, the failure of measurement equipment to record data in the first place, it could be concluded that the validity of the overall results was unaffected. The results reported the 16 resulting datasets.

### 3.1. Correlational Analysis

The results of the correlational analysis for each of the four joint angles (knee, hip, elbow and shoulder) derived from OpenPose in the four different camera viewing angles (front right, front left, back left, back right) compared against the joint angles derived from the marker-based MoCap system are reported in Table 2. The ICC is a measure of reliability and reports to what extent measurements of the same variable (e.g., joint angle) are consistent across different observers, which in this case represent different measurement methods. Excellent ICC values (≥0.9) indicate that the system is reliable in tracking movements with minimal variability across different trials.

Looking at the correlation coefficient, large correlations are reported for all joints using all camera angles, except for the shoulder when using the front right camera. The absolute agreement in terms of ICC(2,1) is excellent for the knee joint for all but the front left camera, which achieves good agreement. The hip joint is in excellent agreement for both cameras in the back, good for the front right and moderate for the front left camera. The agreement for the elbow joint is moderate for all camera views. Closer inspection of the shoulder joint angle reveals moderate agreement for all cameras except for the front right view, which achieves poor agreement. The results of the consistency analysis, in terms of ICC(3,1), show very similar results to ICC(2,1), with slightly higher consistency of the hip joint angle in the front right and front left views, as well as for the shoulder in the front left view and elbow joint angle in the back right view.

In the results, the shoulder flexion/extension angle derived from OpenPose shows only poor to moderate agreement with the marker-based MoCap system for almost all views. The elbow flexion/extension angle shows good agreement and consistency. Mixed results are reported for the hip flexion/extension angle but yield excellent agreement from both cameras viewing from the back. The knee flexion/extension angle is best represented by the OpenPose estimation from most angles; however, the front left view only reaches good agreement.

### 3.2. Systematic Bias Analysis

**Figure 4:**
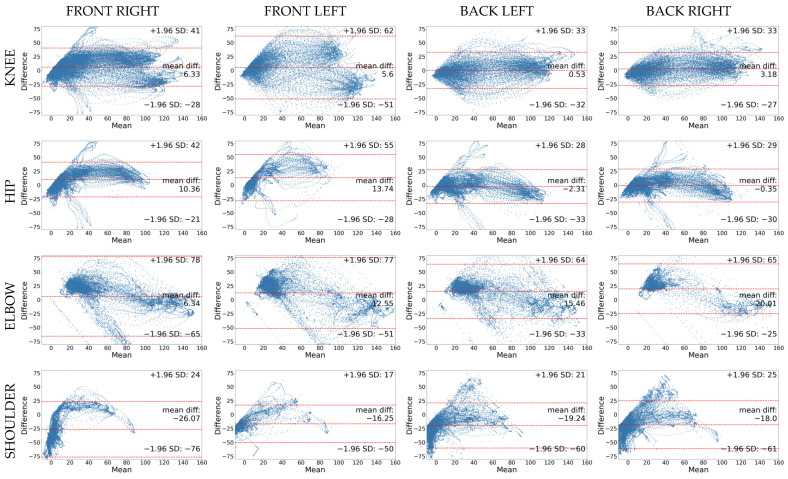
Bland Altman plots for the knee flexion/extension angle (first row), hip flexion/extension angle (second row), elbow flexion/extension angle (third row) and shoulder flexion/extension angle (last row) comparing all 4 camera views (columns) with the marker-based MoCap system. The difference (y-axis) is calculated by subtracting the OpenPose measurement from the Vicon measurement. The mean (x-axis) represents the mean of both measurements. The dashed lines represent the lower and upper limits of agreement (±1.96 standard deviation [SD]) as well as the mean difference (bias). Each measurement of a joint angle is represented as one data point in the point cloud.

Even though the correlation analysis yields good to excellent results for all joints except the shoulder flexion/extension, the Bland Altman analysis reveals deeper insights into the bias and the lower and upper limits of agreement. Bland Altman plots for all joint angles and camera angles are shown in Figure 4.

A Bland Altman plot is a graphical method of analyzing the agreement between two measurement methods. The x-axis represents the mean of the two measurement methods for each observation and the y-axis shows the difference between the methods for each data point. The dashed line in the middle stands for the mean difference or bias between the two methods. If this line is close to zero, it indicates that, on average, the two measurement systems produce similar results; otherwise, the method under investigation either underestimates or overestimates the gold standard. The Bland Altman plots of this study are quite revealing in several ways. First, we can identify systematic biases when looking at the mean differences. Biases range from −0.35° (for the hip joint in the back right view) to −26.07° (for the shoulder joint in the front right view) and are summarized in Table 3. Second, the upper and lower limits of agreement, represented by the top and bottom dashed lines and characterized by ±1.96 standard deviations, show the variance of the calculated joint angles compared to the marker-based joint angles. High limits of agreement suggest poor agreement, while narrow limits indicate good agreement. Points outside these limits are considered outliers. These limits differ for the different joint angles, but are largest for the elbow flexion/extension joint for all camera views. A closer inspection of the scatter of the data points shows that variance increases at bigger joint angles (higher values on the x-axis), which indicate a deviation from the gold standard as the range of motion increases. Systematic scatter of data points, in contrast to random scatter, may indicate a proportional bias. This is in line with the results of the RMSE, which is also presented in Table 3. The RMSE is a measure of precision, which quantifies the deviation from the true value (gold standard). Low values indicate that the system under investigation provides accurate measurements and is able to track movements with minimal deviations from the marker-based MoCap. Over all joint angles and camera views, the average deviation between the OpenPose joint angles and the marker-based joint angles ranges from 15.2° to 36.48°.

**Figure 5:**
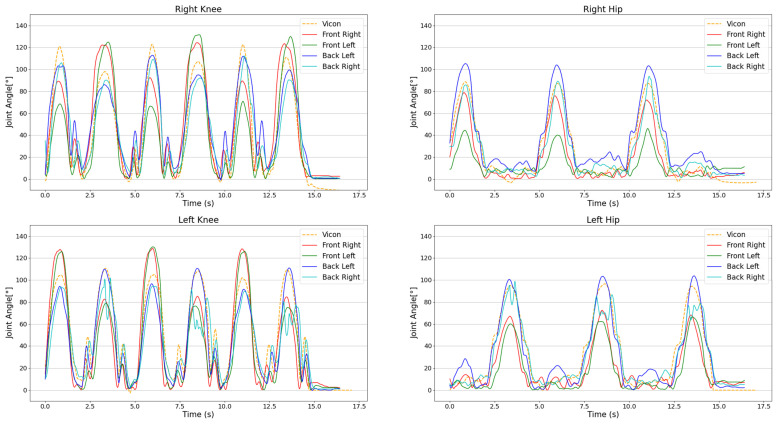
Exemplary plot of right and left knee and hip joint angle curves of all camera views in solid lines and the marker-based MoCap system (Vicon) in a dashed line.

To gain a better impression of this issue, exemplary plots of the right and left knee and hip joint angles of one subject are illustrated in Figure 5. Deviations from the gold standard, especially in the upper ranges of motion, become evident from this example.

**Figure 6:**
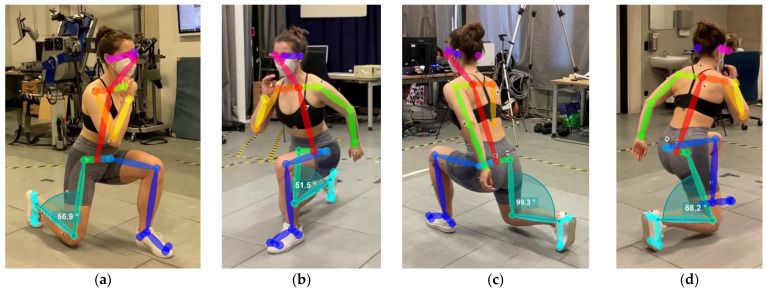
Graphical comparison of knee angles measured across all camera views simultaneously during the turning point of the lunge, which also represents the peak knee angle. (**a**) Front Right: 56.9°. (**b**) Front Left: 51.5°. (**c**) Back Left: 99.3°. (**d**) Back Right: 68.2°.

Deeper investigation of the OpenPose video data reveals no estimation errors but rather the issue of perspective when calculating 2D joint angles, as demonstrated in Figure 6. Looking at the different perspectives one by one, there seems to be no error in the estimation of the different keypoints; hence, the calculation of the different joints seems correct. However, when comparing all views at once, it becomes evident that the camera viewing angle impacts the joint angle estimation tremendously, especially at the full range of motion.

### 3.3. Analysis of Peak Joint Angles

To quantify the influence of the camera viewing angle on the full range of motion, an analysis of the peak values is presented in the following. The mean joint angle values and the corresponding standard deviation of the four joint angles (knee, hip, elbow, shoulder) for the gold standard (Vicon) and all four camera angles is presented in Table 4. The standard deviation is explained by the individual range of motion of each of the participants; however, it would be expected to be similar over all measurement systems.

From the mean joint angle values at the peak knee flexion angle, we can estimate systematic differences in the different views but still cannot quantify the deviations. The absolute deviations of each joint angle for each camera from the gold standard averaged over all subjects are summarized in Table 5.

From Table 5, we can see the average deviation of each joint angle averaged over all camera views as well as the average deviation of all joint angles for each of the four camera views. The highest mean deviation from the gold standard was found in the front left camera view at 23.8°, whereas the other cameras report similar mean deviations from the gold standard, ranging from 14.1° to 14.9°. Closer inspection of the joint angles reveals that the worst performing is the estimation of the left knee joint angle with a mean deviation of 20.1° over all camera views, and best performing is the right shoulder joint angle, with a mean deviation of 12.2°. In order to facilitate a more precise categorization of the absolute deviations, Table 6 illustrates the mean percentage deviation from the peak value of the gold standard.

**Figure 7:**
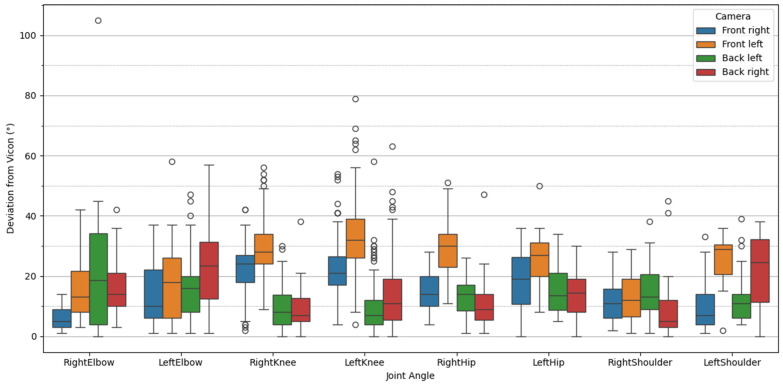
Boxplot of the deviations from Vicon (°) for all joint angles and camera viewing angles. The boxes represent the data between the first and the third quartiles. The horizontal line within the box indicates the median, while the whisker extends to data points that fall within 1.5 times the interquartile range. Outliers are represented as circles.

The highest percental deviations of peak values are found for the left shoulder in the front left camera (49%) and back right camera (42%). The lowest percental deviations are to be seen in the elbow joint with a mean deviation of 14% over all camera views. When comparing the different camera viewing angles, it becomes evident that the front left camera shows the highest mean percental deviation of 27%, whereas all other perspectives show similar results of 17–18%. When excluding the worst-performing joint, which is the shoulder, both back-viewing cameras show the lowest mean deviations of 13%. A graphical representation of the deviations at the peak knee flexion is shown in Figure 7.

From this boxplot, we see the highest deviations in the front left camera, shown in orange. Mean deviations >20° are reported for the knee joint (right and left), hip joint (right and left) and left shoulder. For the front right camera, portrayed in blue, mixed results can be found. This view performs best in the estimation of the right elbow and left shoulder but fails at the knee and hip joint. The analysis of the back left camera, depicted in green, shows <20° for all joints, but indicates surprisingly high variance for the right elbow. The back right camera, shown in red, achieves comparatively good results for the knee (left and right), hip (left and right) and right shoulder, but high deviations for the left elbow and left shoulder.

## 4. Discussion

The objective of this study is to quantify the impact of the camera angle on the accuracy of OpenPose joint angle estimation and to provide insights into the potential inaccuracies that may arise when deviating from the standard camera angles (frontal and sagittal). Therefore, we investigated four joint angles (knee, hip, elbow, shoulder) during front lunges performed by 20 participants and compared them against a marker-based MoCap system. Joint angles were calculated from keypoints estimated using OpenPose from video data recorded from four different camera angles.

Several studies have examined the validity of OpenPose joint angle estimation [5,22,23,24,26,27,28,29,34,43,44]. ICCs were reported as good for the knee (ICC = 0.8) and poor for the hip (ICC = 0.37) during bilateral squats [26]. Furthermore, a study investigating running and walking on a treadmill stated similar results for the hip (ICC = 0.43–0.86) and knee (ICC = 0.52–0.91) for different walking speeds [28]. Another study examined the validity of OpenPose during gait with orthosis and found that the ICC values for hip and knee kinematics were good to excellent (ICC = 0.60–0.98) [43]. Those findings are partly in line with our finding, where the ICC of the knee joint was reported as good to excellent (ICC = 0.86–0.96), whereas the hip joint achieved a higher correlation (ICC = 0.74–0.91) in our study compared to the literature. When comparing the individual camera views, it becomes evident that the front left viewing point only has moderate to good agreement (ICC = 0.72–0.86), while all others show mostly good to excellent agreement for all joint angles except the shoulder joint, which has poor to moderate agreement for all camera views. This is a first indication that the front left view is not ideal for this type of movement. Intraclass correlations are an indication of how strongly two measurements resemble each other. High correlations mean the joint angle curves show similar waveforms and trends, which is essential in the validity analysis.

With regard to the RMSE, one study examining running kinematics obtained RMSE values of 4.95–5.92° for the hip and 7.45–7.81° for the knee, depending on the running speed [29]. This is in contrast to the RMSE values reported in our study, which ranged from 15.50° to 29.44° for the knee and 15.20° to 25.25° for the hip angle.

Nevertheless, the correlational analysis and RMSE do not provide information on a potential systematic bias between the measurement systems. Bland Altman analysis can provide such insights [45]. Previous studies reported biases of 4–8° for the knee and hip joints during cycling [5], approximately 5–8° for the knee and hip joints during bilateral squats [26] and biases ranging from −1.9° to 2.1° for knee and −3.0° to 15.2° for hip joints in walking, running and cycling [44]. Consistent with the literature, this research found biases for the knee joint ranging from 0.53° to 6.33° for the different camera views. The bias for the hip joint ranged from −2.31° to 13.74°, which is in accordance with previous findings. In contrast, the shoulder joint shows comparatively high negative biases ranging from −16.25° to −26.07°. On the other hand, looking at the different camera angles, the back left camera view achieves the lowest bias of −1.39° overall. However, this result should be treated with caution since the average bias of the elbow joint for the back left camera is 15.46° and the bias for the shoulder is −19.24°, which compensate for each other in the overall bias. The same is true for the back right camera view, where the overall bias of −1.55° is mainly the result of a high positive bias of the elbow (20.01°) and a high negative bias of the shoulder (−18.00°). Comparing the overall biases is therefore difficult, and systematic bias should be investigated at a joint angle level for each of the camera views. Different strategies could be applied to help reduce the observed systematic bias. We propose incorporating biomechanical constraints, such as restricting joint angles to anatomical limits. Furthermore, algorithms could improve joint angle estimation by applying penalties for unrealistic angles or significant deviations in joint angles between consecutive frames. In addition, optimization algorithms might help overcome non-optimal perspectives.

Additionally, the overall prediction error, in terms of RMSE, can provide insights into both the systematic bias and the variance of the deviations between the OpenPose joint angle and the gold standard. RMSE values of 5.1° for the knee and 5.6° for the hip joint are reported in the literature for walking, running and cycling [44]. Similarly, RMSE values of 6.9° and 10.2° for the knee and hip, respectively, were found in gait analysis [24]. These values reported in the literature are far below the RMSE values observed in this study. The RMSE for the knee ranges from 15.50° to 29.44°, and for the hip joint, it is from 15.20° to 25.25°. The RMSE values for the elbow and shoulder joints are even higher (23.63–37.10°) over all camera views. This is an indication of a high variance of deviations in the data. This can also be seen in the Bland Altman plots in Figure 4. Closer inspection of the knee joint, for example, shows that even though the bias is relatively low, the upper and lower limits of agreement range from −51° to 62° for the front left view and between −27° and 38° for all other camera views. This is another indication that the front left view produces inaccurate results.

The results of the Bland Altman analysis highlight the importance of not only looking at the correlational analysis but also checking for systematic biases in the measurement systems. A closer analysis of the peak values may provide more insight into those deviations. From Figure 5, it can be seen that the highest deviations occur during the peaks of the movement when the joints are in the full range of motion. When looking at the mean absolute deviations from the gold standard (Table 5), it becomes clear that the front left camera shows high deviations in the knee (30.2–33.3°) and hip (25.2–30.0°). This results in an overall mean absolute deviation of 23.8° over all joints for the front left camera, which is significantly higher than the other perspectives (14.1–14.9°). These findings are contrary to previous studies, which have demonstrated average absolute errors of 3.7° for the hip joint and 5.1° for the knee joint over the course of a gait cycle [23].

However, for an interpretation of the deviations’ severity, a closer look should be taken at the percental deviations (Table 6). For example, a deviation of 25° in the shoulder joint results in a percental deviation of 49%, compared to the hip joint, where a deviation of 25.2° only results in a percental deviation of 27%, since the mean peak value of the shoulder is at 52.8° and that for the hip is at 77.8°. In the overall evaluation, the front left camera again achieves the worst results (27% mean percentage deviation) for all camera views. In terms of joints, the shoulder joint is the worst represented, with a mean percentage deviation of 29%.

In order to comprehend why there is such a big difference in peak joint angle values for the different camera perspectives, a closer look at the different views and keypoints detected by OpenPose can provide a deeper understanding. In Figure 6, we can see one subject during the turning point of the lunge, which also represents the peak knee angle. What is apparent from looking at it is the big difference in the knee joint angle. The knee joint angles are calculated using the left/right hip keypoint, the left/right knee keypoint and the left/right ankle keypoint (as described in Table 1). Therefore, a possible explanation for the differences is the detected keypoints that differ from view to view. Looking at the ankle keypoint, this should be relatively consistent for all views since the ankle is quite a dominant anatomical landmark. The hip joint, however, is much more difficult to estimate correctly since it has an offset from the skin to the joint center. Even though the subject wears tight-fitting shorts, it is not easy to determine where the anatomical hip joint center is located. This effect is reinforced by the fact that in some camera perspectives, the hip is seen from the anterior, and for some, in the posterior or even a sagittal viewpoint, or somewhere in between. The same is true for other joints or keypoints, such as the neck (as defined as an OpenPose keypoint), which is used for calculating some of the joint angles investigated. Slight deviations in keypoint estimation can have a big effect on the calculation of the corresponding joint angle. Furthermore, as video-based MoCap fully relies on the image, it is of particular importance that the joints of interest are actually visible to the camera. When taking a look at Figure 6, it becomes apparent that the front left camera had the worst view of the right leg—so it is not really surprising that this view performed worst. Occlusions can occur in any of the perspectives; however, they appear rather seldom in the lower body in the perfect sagittal or frontal perspectives, which indicates those to be superior when investigating lower body joints. Especially the arms are prone to occlusions by the torso, which makes an accurate estimation of the elbow and shoulder joints challenging. This issue remains for video recordings from a sagittal view but is rather unusual for the frontal camera view. Those findings are in line with previous research [46].

Which is the best-performing perspective? The graphical exploration of the boxplots in Figure 7 provides insights into the performance of each of the camera views on each of the joint angles. It becomes evident that again the front left camera produces high inaccuracies on both knee joints, both hip joints, the left shoulder and the left elbow. This raises the question of whether the near-side joint angles are more prone to inaccurate detection. This can be confirmed for all joint angles for the front left camera except for the hip joint, where the far-side (right) hip joint angle estimation performs slightly worse than the near-side (left). However, the deviation results of the front right camera do not support this hypothesis, since there is no clear pattern concerning far-side and near-side joint angles identifiable. The right (near-side) elbow achieves lower deviations than the far-side elbow. The same is true for the hip, but the opposite is true for the knee and shoulder joints. On the other hand, for both back-view cameras, this proposal does not apply. The near-side joint angles are better estimated in both back-viewing cameras for all joints. In general, the back-viewing cameras demonstrate lower deviations, indicating that the back views are most suitable for this type of exercise estimation. This assumption is supported by the results of the percentage deviations (in Table 6), which are lowest (13%) for the two rear cameras when the shoulder is excluded. This phenomenon could be attributed to a better visibility of the major key points, particularly the hips and shoulders, where occlusions are less prominent in the back view. The center of the body, i.e., the right, left and mid hip in combination with the neck, are predominantly used in the calculation of the majority of the joint angles. These keypoints exhibit better visibility in rear-view perspectives and are not obscured by the limbs.

A few limitations of this study should be noted. First, we compared 3D joint angles of a marker-based MoCap system against joint angles derived and calculated in 2D planes. Joint movements in different planes, such as abduction/adduction and flexion/extension of the hip or shoulder, for example, as well as rotations, are difficult to consider separately in two-dimensional space, as they usually overlap. However, this also applies for all other studies evaluating the performance of Human Pose Estimation Algorithms against a marker-based MoCap system, but might have a bigger overlapping effect in 45°-degree angles compared to sagittal and frontal viewpoints. Next, it should be noted that we investigated each camera view separately. The combination of keypoints from multiple camera views has the potential to yield 3D joint angle data and may correct for some of the errors and deviations. However, the primary goal was to assess the accuracy of videos that may have been recorded in a home or clinical setting, where it is assumed that only a single device was used. Therefore, the analysis was limited to each camera view individually. Yet, research in the field of human pose estimation has brought significant advances since the first introduction of OpenPose. Techniques and algorithms now exist that are capable of elevating pose estimation from 2D into 3D [47,48,49]. In addition, HPE algorithms for the extraction of 3D pose from 2D images have been developed [16]. One example of such an algorithm is MediaPipe [50]. In the long term, the future of HPE is therefore considered to be in 3D analysis rather than 2D analysis. Finally, with only one exercise being investigated in this research, caution must be applied, as the findings might not be applicable to other types of movements.

Overall, it is as yet unclear whether these results can be generalized to other types of movements and exercises. This question can be addressed in future research, which will verify its validity for other types of movements. While our study focused on a simple movement, we believe that further exploration of more complex motions could be beneficial. Specifically, complex movements with higher degrees of freedom, rotations, highly dynamic motions or movements involving rare body poses, such as a handstand or lying on the floor, may provide additional insights. It may also be beneficial to provide recommendations on camera viewing angles depending on the exercise in question. We furthermore encourage the use of multiple camera combinations to investigate this effect. A comparison with the standard 2D recording angles (sagittal and frontal) may also prove beneficial in future research. Furthermore, it would be advantageous to correct the data for fixed or proportional biases and explore the discrepancy between the true values and the data obtained by OpenPose using this offset.

## 5. Conclusions

Using OpenPose in biomechanics research can help overcome some of the drawbacks of conventional marker-based MoCap systems, especially in terms of time and costs, making it a valuable tool, especially in home training or clinical environments, where only one camera, such as, for example, a standard smartphone, is needed. However, the validity of kinematics derived from this human pose estimation algorithm needs to be assessed carefully before recommending the use of those in diverse applications. Therefore, we investigated the influence of the camera viewing angle on OpenPose validity in joint angle estimation.

The analysis of correlation indicates the feasibility of using camera angles other than the two standard views by showing good agreement for hip and knee joints for all camera viewing angles. Furthermore, Bland Altman analysis confirmed these findings by demonstrating similar biases for knee and hip joints as reported in the literature. However, the RMSE revealed high variance in the deviations from the gold standard, indicating inaccuracies arising from camera views other than frontal or sagittal. These results were confirmed by the analysis of peak angle values. For the shoulder joint, no satisfying results can be reported for any of the camera views. Nonetheless, this research has also demonstrated that the cameras filming the subject from the back resulted in the lowest percental deviations.

A proper camera positioning and setup are crucial for optimizing the performance of OpenPose. The following recommendations are proposed based on the findings of this study. In instances where sagittal or frontal camera angles are anticipated to fall short in capturing the complete range of motion of the body segments under investigation, we suggest the use of back-viewing camera angles rather than front-viewing camera angles. However, the validity of a specific camera angle for a specific movement must first be assessed, since the results of this study demonstrate that the camera angle does indeed have an impact on the validity of pose estimation. In the context of practical applications, it is proposed that camera angles should be used which produce the fewest occlusions of major keypoints while still capturing the primary movement plane, to optimize the estimation of OpenPose. It is vital to ascertain that the subject occupies the majority of the frame without being cut off and to ensure that the whole of the measurement space is captured; for instance, ensuring sufficient height for a jump if such movement is of interest. We firmly encourage testing of the setup and making adjustments as required to ensure that no occlusions of the major keypoints occur during the movement before selecting one camera angle.

This study contributes to the understanding of the feasibility and validity of using OpenPose for joint angle estimation in biomechanics research and its potential limitations. The evidence from this study supports the idea of using camera angles other than those that are standard but also stresses the necessity of investigating individual movements closely before suggesting certain camera angles for users in home training or practitioners in clinical settings.

## Figures and Tables

**Table 1:** Definition and calculation of joint angles using keypoints extracted from OpenPose.

Joint	Angle
Knee Flexion/Extension	The angle between the keypoints RHip, RKnee and RAnkle.
Hip Flexion/Extension	The angle between the straight line from RHip to RKnee and the straight line from MHip to Neck.
Shoulder Flexion/Extension	The angle between the straight line from RShoulder to RElbow and the straight line from MHip to Neck.
Elbow Flexion/Extension	The angle between the keypoints RShoulder, RElbow and RWrist.

Joint angles for the left side are likewise.

**Table 2:** Correlation coefficient (r) and intraclass correlation coefficient [ICC (2,1) and ICC (3,1)] for each joint angle, comparing each camera viewing angle against the gold standard.

Joint	Front Right			Front Left			Back Left			Back Right		
	r	ICC (2,1) [95% CI]	ICC (3,1) [95% CI]	r	ICC (2,1) [95% CI]	ICC (3,1) [95% CI]	r	ICC (2,1) [95% CI]	ICC (3,1) [95% CI]	r	ICC (2,1) [95% CI]	ICC (3,1) [95% CI]
Knee	0.91 *	0.95 * [0.95–0.95]	0.95 * [0.95–0.95]	0.76 *	0.86 * [0.85–0.87]	0.86 * [0.86–0.87]	0.91 *	0.95 * [0.95–0.95]	0.95 * [0.95–0.95]	0.93 *	0.96 * [0.95–0.96]	0.96 * [0.96–0.96]
Hip	0.87 *	0.88 * [0.71–0.93]	0.91 * [0.91–0.91]	0.75 *	0.74 * [0.47–0.85]	0.80 * [0.79–0.81]	0.89 *	0.94 * [0.93–0.94]	0.94 * [0.94–0.94]	0.88 *	0.94 * [0.94–0.94]	0.94 * [0.94–0.94]
Elbow	0.65 *	0.75 * [0.73–0.76]	0.75 * [0.75–0.76]	0.76 *	0.80 * [0.73–0.85]	0.82 * [0.81–0.83]	0.80 *	0.83 * [0.64–0.90]	0.87 * [0.87–0.88]	0.89 *	0.84 * [0.43–0.93]	0.90 * [0.90–0.91]
Shoulder	0.36 *	0.28 * [0.08–0.50]	0.44 * [0.43–0.45]	0.79 *	0.72 * [0.14–0.87]	0.83 * [0.82–0.84]	0.63 *	0.59 * [0.04–0.78]	0.73 * [0.72–0.73]	0.58 *	0.55 * [0.11–0.74]	0.67 * [0.66–0.68]

* *p*-value < 0.001. Abbreviations: CI, confidence interval; ICC, intraclass correlation coefficient. ICC interpretations of poor, moderate, good and excellent are colored in red, orange, yellow and green, respectively.

**Table 3:** Root mean square error (RMSE [°]) and bias [°] for each joint angle, comparing each camera viewing angle against the gold standard.

Joint	Front Right		Front Left		Back Left		Back Right	
	RMSE [°]	Bias [°]	RMSE [°]	Bias [°]	RMSE [°]	Bias [°]	RMSE [°]	Bias [°]
Knee	18.23	6.33	29.44	5.60	16.66	0.53	15.50	3.18
Hip	19.00	10.36	25.25	13.74	15.72	−2.31	15.20	−0.35
Elbow	37.10	6.34	34.96	12.55	29.35	15.46	30.35	20.01
Shoulder	36.48	−26.07	23.63	−16.25	28.31	−19.24	28.46	−18.00
**Overall**	**31.79**	**−5.89**	**29.91**	**5.71**	**23.38**	**−1.39**	**22.15**	**−1.55**

**Table 4:** Mean joint angle values (°) ± standard deviation (°) at peak knee flexion angle over all participants.

Joint	Vicon	Front Right	Front Left	Back Left	Back Right
Knee right	111.2 ± 12.6	107.8 ± 19.7	107.0 ± 25.7	100.8 ± 12.6	107.2 ± 11.8
Knee left	114.0 ± 15.9	109.4 ± 18.0	103.6 ± 28.5	109.2 ± 15.3	100.4 ± 14.8
Hip right	94.8 ± 7.3	81.0 ± 10.9	67.8 ± 13.1	107.3 ± 10.7	104.9 ± 13.6
Hip left	93.0 ± 5.8	77.8 ± 14.3	69.2 ± 11.6	109.6 ± 12.3	105.5 ± 13.0
Elbow right	121.4 ± 14.8	122.0 ± 16.2	133.2 ± 32.1	122.0 ± 23.2	137.4 ± 17.4
Elbow left	115.5 ± 16.5	121.0 ± 32.7	130.5 ± 20.4	129.2 ± 26.0	116.1 ± 20.0
Shoulder right	50.7 ± 17.5	56.4 ± 22.2	46.3 ± 22.6	58.8 ± 17.9	58.4 ± 22.1
Shoulder left	50.7 ± 15.3	52.8 ± 21.9	46.1 ± 20.2	62.2 ± 20.8	51.7 ± 21.8

**Table 5:** Mean absolute deviations from the gold standard (°) at the knee peak flexion angle over all participants.

Joint	Front Right	Front Left	Back Left	Back Right	Mean	Overall
Knee right	22.7	30.2	9.4	8.9	17.8	19.0
Knee left	23.1	33.3	9.7	14.3	20.1	
Hip right	15.3	30.0	13.2	10.9	17.4	17.7
Hip left	18.4	25.2	15.2	13.7	18.1	
Elbow right	5.8	15.7	21.8	15.7	14.7	16.3
Elbow left	12.9	17.7	16.3	24.2	17.8	
Shoulder right	11.7	13.4	14.5	9.3	12.2	14.7
Shoulder left	9.6	25.0	12.7	21.5	17.2	
Overall	14.9	23.8	14.1	14.8		

Color codes: dark red indicates the highest values of deviation, whereas dark green is an indication of the lowest deviation. All color gradations from red to green are classified in between.

**Table 6:** Mean percentage deviations from the gold standard at the knee peak flexion angle over all participants.

Joint	Front Right	Front Left	Back Left	Back Right	Mean
Knee right	20%	27%	8%	8%	**17%**
Knee left	20%	29%	9%	13%	
Hip right	16%	32%	14%	11%	**19%**
Hip left	20%	27%	16%	15%	
Elbow right	5%	13%	18%	13%	**14%**
Elbow left	11%	15%	14%	21%	
Shoulder right	23%	26%	29%	18%	**29%**
Shoulder left	19%	49%	25%	42%	
**Mean**	**17%**	**27%**	**17%**	**18%**	
**Mean excl. shoulder**	**15%**	**24%**	**13%**	**13%**	

Color codes: dark red indicates the highest values of deviation, whereas dark green is an indication of the lowest deviation. All color gradations from red to green are classified in between.

## Data Availability

The raw data supporting the conclusions of this article will be made available by the authors on request.
